# Fracture Morphology and Extraction-Related Femoral Damages in Short Versus Standard Cementless Stems: Analyses of Vancouver B2 Fractures and Well-Fixed Nonfracture Revisions

**DOI:** 10.1016/j.artd.2026.102150

**Published:** 2026-10-09

**Authors:** Dominic Simon, Gautier Beckers, Florian Pachmann, Amir Koutp, Salome Hagelstein, Eduardo Suero, Boris M. Holzapfel, Lukas Leitner

**Affiliations:** a Department of Orthopaedics and Trauma Surgery, Musculoskeletal University Center Munich (MUM), University Hospital, LMU Munich, Munich, Germany; b Univ.-Klinik für Orthopädie und Traumatologie, Medizinische Universität Graz, Austria

**Keywords:** Short stem, Standard stem, Revision total hip arthroplasty, Vancouver B2, Periprosthetic femoral fracture, Stem extraction, Osseointegration

## Abstract

**Background:**

Short-stem total hip arthroplasty is increasingly used because of its bone-preserving, minimally invasive concept. Revision may occur in two fundamentally different situations: Vancouver B2 periprosthetic femoral fracture (PFF) with an unstable stem or removal of a well-fixed osseointegrated stem for a nonfracture indication. This study evaluated whether stem design is associated with distinct fracture morphology and extraction-related femoral damage.

**Methods:**

This retrospective two-center cohort included 102 revision total hip arthroplasties performed between 2021 and 2025: standard-stem Vancouver B2 PFF (n = 44), short-stem Vancouver B2 PFF (n = 12), standard-stem nonfracture revision (n = 27), and short-stem nonfracture revision (n = 19). Primary analysis compared fracture localization and morphology between Vancouver B2 cohorts; secondary analysis compared intraoperative fractures and extraction-related reconstruction requirements between well-fixed nonfracture cohorts. Perioperative outcomes were reported descriptively.

**Results:**

In Vancouver B2 PFF, short stems more frequently involved the greater trochanter (58.3%), lesser trochanter (33.3%), calcar (41.7%), and lateral cortex (58.3%) vs standard stems (40.9%, 9.1%, 13.6%, and 31.8%, respectively). During well-fixed nonfracture revision, intraoperative fracture occurred in 7/19 (36.8%) short-stem vs 4/27 (14.8%) standard-stem revisions. Cerclage fixation was required in 10/19 (52.6%) vs 4/27 (14.8%), and modular diaphyseal reconstruction in 9/19 (47.4%) vs 8/27 (29.6%), respectively.

**Conclusions:**

Short and standard cementless stems showed different femoral involvement in Vancouver B2 PFF. Separately, removal of well-fixed short stems was frequently associated with extraction-related proximal femoral damage and additional fixation. These findings do not establish universal superiority of either design but emphasize considering clinical scenario, fixation pattern, and stem geometry independently when planning revision THA.

## Introduction

The use of short stems in total hip arthroplasty (THA) has increased in recent years, reflecting growing interest in this design [1].These implants offer potential advantages such as minimally invasive surgery, bone preservation, potential facilitated revision, and reduced stress shielding [[2], [3]–4].

Furthermore, short stems are designed to promote proximal load transfer and bone remodeling by facilitating osseointegration, with most stems coated with hydroxyapatite [5,6].

Therefore, long-term outcomes and implant survivorship are anticipated to be comparable or superior to those of standard stems, with 5-year revision rates reported as low as 1.8%. However, these findings should be interpreted with caution, as younger and fitter patients are more frequently selected for short-stem implantation, introducing a potential selection bias [7,8].

Recent investigations assessing the risk of postoperative periprosthetic femoral fracture (PFF) following cementless THA using short vs straight femoral stems have yielded inconclusive results. While short-stem THA has been associated with a lower incidence of Vancouver type A PFF, the occurrence of Vancouver type B fractures appears comparable between the two stem designs [9].

Nevertheless, strong metaphyseal press-fit with rigid biological fixation could be associated with specific fracture patterns and potential challenges during stem revision, as loading patterns and bone mineral density values differ between short and standard stems [[10], [11]–12].

Furthermore, as the number of hip arthroplasties continues to increase, the incidence of periprosthetic fractures is expected to rise proportionally, potentially resulting in a substantial clinical and economic burden in the future [9,13,14].

The primary objective was to compare fracture localization and morphology in Vancouver B2 PFFs around short vs standard cementless stems. The secondary objective was to compare extraction-related femoral damage and reconstruction requirements during removal of well-fixed, osseointegrated short vs standard stems for nonfracture indications.

## Material and methods

### Data collection and study population

We performed a retrospective cohort study across two academic centers, including all patients who underwent THA revision between January 1, 2021, and July 30, 2025, either for a Vancouver B2 PFF requiring stem revision or for a nonfracture indication requiring removal of a well-fixed, osseointegrated cementless femoral stem. Six senior consultant surgeons from the two centers contributed cases. All routinely performed complex revision THA and management of PFFs.

For nonfracture revisions, osseointegration of the femoral stem was assessed using preoperative radiographs and intraoperative mechanical assessment. Radiographic criteria included the absence of progressive radiolucent lines (>2 mm), subsidence, migration, or varus/valgus tilt and the presence of findings compatible with biological fixation such as spot welds, cortical hypertrophy, or trabecular bridging. Definitive intraoperative confirmation consisted of solid axial and rotational fixation during attempted extraction. In contrast, Vancouver B2 cases were defined by a periprosthetic fracture around or just distal to the stem with mechanical stem instability and were therefore analyzed separately from the well-fixed cohort.

Patients with incomplete medical records, revision procedures without femoral stem extraction, or fractures classified as Vancouver A, B1, B3, or C, as treated with open reduction and internal fixation, were excluded [5]. Among 305 patients identified in our institutional prospective revision arthroplasty database, 102 met the inclusion criteria and were included in the final analysis. Data were obtained from patients’ electronic medical records. The inclusion and exclusion process is illustrated in Figure 1.

**Figure 1:**
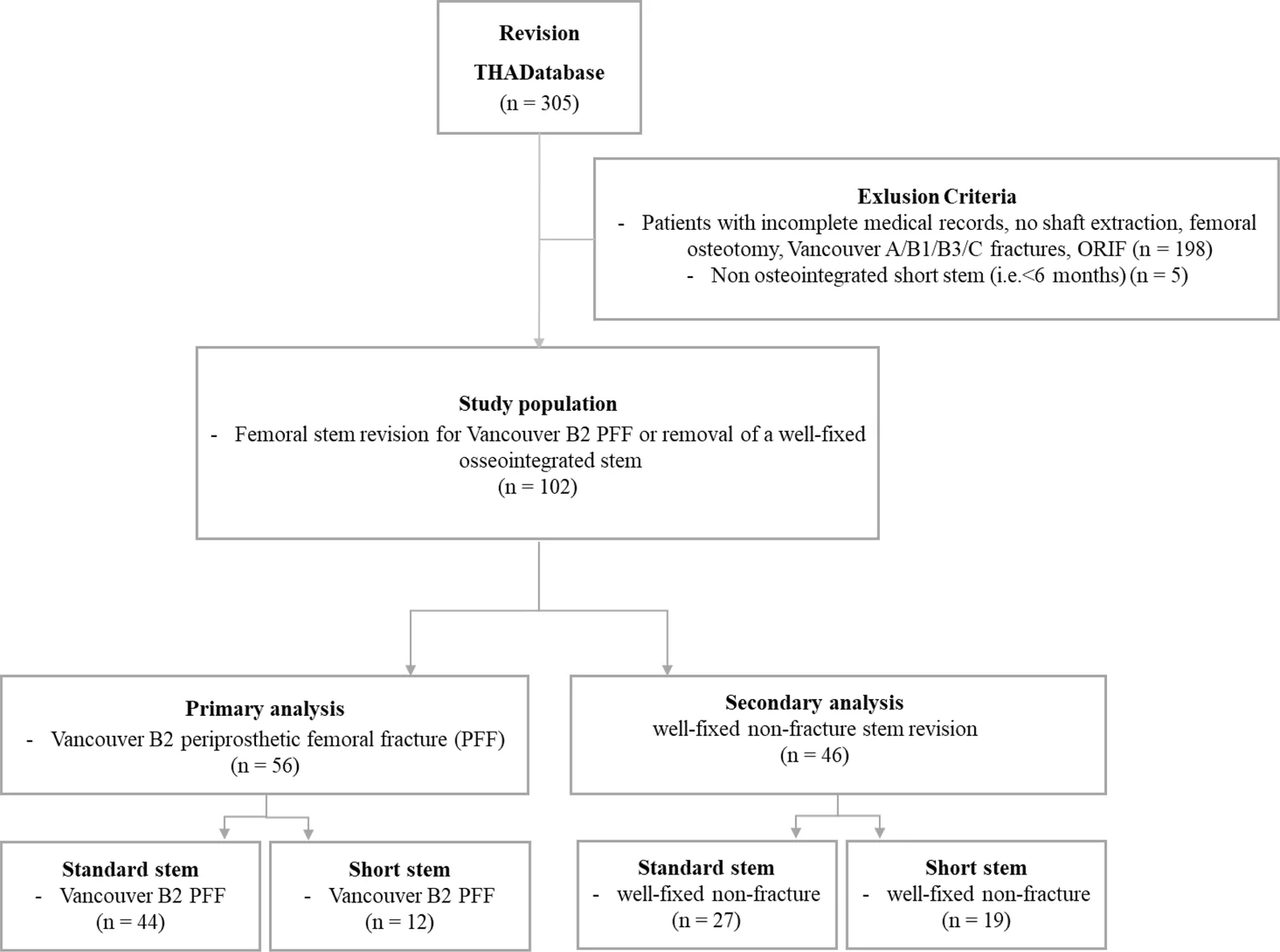
Flowchart showing assignment of the 102 included patients to four mutually exclusive cohorts according to clinical scenario (Vancouver B2 PFF vs well-fixed nonfracture revision) and stem design (standard vs short). ORIF, open reduction and internal fixation; PJI, periprosthetic joint infection.

Although there is currently no universal consensus on the definition of a short stem, femoral components in short stem THA are commonly described as implants designed for predominant metaphyseal fixation with a total length (center of head to prosthesis tip) of approximately ≤120 mm. While this length-based threshold is frequently used, several authors emphasize that the defining characteristic of short stems lies in their metaphyseal, rather than diaphyseal, anchorage, and that a purely dimensional definition may not adequately reflect the heterogeneity of contemporary stem designs [15].

In this study, all short stems corresponded to type 4 according to the classification proposed by Khanuja et al [16], where femoral stems are categorized based on their length and fixation concept rather than fracture behavior. Type 4 stems are characterized by predominant metaphyseal anchorage with minimal or no diaphyseal fixation, aiming to preserve proximal femoral bone stock and promote physiological load transfer. In contrast, the Vancouver classification is a fracture-oriented system that categorizes PFFs based on fracture location, stem stability, and bone stock quality, and does not account for implant design or fixation principles. Thus, the Khanuja classification was used in the present study to define stem design, while the Vancouver classification was applied exclusively for fracture assessment and revision indication.

Femoral stems were classified as either standard or short. There were 71 patients with a standard stem (41 women; mean age, 75.9 ± 11.6 years) and 31 with a short stem (12 women; mean age, 69.1 ± 14.6 years), with stem explantation occurring at a mean interval of 6.2 ± 5.3 years after implantation.

Patients were assigned to four mutually exclusive cohorts according to clinical scenario and stem design: (1) well-fixed standard-stem nonfracture revisions (n = 27), (2) well-fixed short-stem nonfracture revisions (n = 19), (3) standard-stem Vancouver B2 PFF revisions (n = 44), and (4) short-stem Vancouver B2 PFF revisions (n = 12). Comparisons were restricted to stem design within the same clinical scenario: short vs standard stems for Vancouver B2 PFF and short vs standard stems for well-fixed nonfracture revision. The cohort structure is shown in Figure 1 and the demographic characteristics are summarized in Table 1.

**Table 1:** Patient demographics of the 4 nonoverlapping cohorts: well-fixed nonfracture revisions and Vancouver B2 periprosthetic femoral fracture revisions, stratified by standard vs short stem.

	All	Standard-stem well-fixed nonfracture	Standard stem Vancouver B2 PFF	Short-stem well-fixed nonfracture	Short-stem Vancouver B2 PFF
	(n = 102)	(n = 27)	(n = 44)	(n = 19)	(n = 12)
Mean age (y)	73.9 ± 12.9	69.1 ± 9.5	**80.1 ± 10.8**^a^**0.04**	**60.9 ± 9.7**^b^**<0.01**	**82.3 ± 10.9**^a^**0.03**
Mean BMI (kg/m^2^)	26.2 ± 5.0	27.2 ± 5.0	25 ± 4.5	**28.6 ± 5.3**	24.9 ± 4.9
Gender					
Female	53 (52%)	13 (48.2%)	28 (63.6%)	7 (36.8%)	5 (41.7%)
Male	49 (48%)	14 (51.8%)	16 (36.4%)	12 (63.2%)	7 (58.3%)
ASA score	2.7 ± 0.7	2.7 ± 0.7	2.7 ± 0.7	2.4 ± 0.6	2.9 ± 0.5
CCI	2.29 ± 1.52	2.28 ± 1.54	2.23 ± 1.43	2.05 ± 1.79	2.91 ± 1.45
Anticoagulants	40/102 (39.2%)	7/27 (25.9%)	**23/44 (52.3%)**	4/19 (21.1%)	**6/12 (50%)**
Implant age (months)	74 ± 64	52 ± 53	**113 ± 69**^a^**0.04**	49 ± 65	**119 ± 42**^a^**0.03**

BMI, body mass index; CCI, Charlson Comorbidity Index; ASA, American Society of Anesthesiologists.

Bold represents statistical significance.

a Represents statistical significance, *P* < .05.

b Represents statistical significance, *P* < .01.

Revision procedures were performed through lateral, anterolateral, or direct-anterior approaches according to previous exposure, implant position, fracture morphology, and surgeon preference. This heterogeneity reflects routine practice at two high-volume revision centers and was considered when interpreting the results. In the well-fixed nonfracture cohort, stem extraction followed a stepwise escalation strategy beginning with circumferential proximal release and dedicated extraction instruments. If secure axial or rotational mobilization could not be achieved, additional exposure such as cortical windowing or extended trochanteric osteotomy was used when judged necessary to reduce uncontrolled fracture propagation.

The decision to extend the femoral exposure was based on intraoperative stem fixation, resistance to extraction, existing cortical compromise, and the anticipated risk of iatrogenic fracture. Extraction technique and the need for additional femoral exposure were documented and were interpreted as features of revision complexity rather than as standardized interventions applied uniformly to all patients [17].

### Data collection

Variables included age, sex, body mass index, incision-to-suture time, estimated blood loss, Charlson Comorbidity Index, American Society of Anesthesiologists classification and surgical and perioperative complications using the Clavien–Dindo Classification. The Clavien–Dindo classification is a standardized system used to grade postoperative complications based on the severity of the intervention required, ranging from minor deviations from the normal postoperative course (grade I) to life-threatening complications or death (grades IV–V). Preoperative hemoglobin levels were assessed, as well as on postoperative day 1 and day 3. Perioperative blood loss was estimated by measuring blood volume in the suction drains and counting and estimating the blood-soaked gauzes. Estimated blood loss was used as a surrogate parameter for surgical invasiveness and procedural complexity. However, this measure is inherently imprecise and subject to inter-surgeon and intraoperative variability. Accordingly, differences in estimated blood loss should be interpreted with caution, and conclusions drawn from this parameter are considered supportive rather than definitive. A restrictive transfusion strategy (hemoglobin <7 g/dL or symptomatic anemia with hemoglobin >7 g/dL) was applied for allogenic red blood cell transfusions. In each revision, the requirement for cerclage wiring was assessed and documented whenever further stabilization of the stem fixation zone was needed. Cerclages were applied either to stabilize and approximate cortical fracture fragments resulting from stem extraction or prophylactically to prevent further fracture propagation in the presence of metaphyseal destruction or cortical compromise during revision surgery.

Patient demographics and perioperative data are summarized in Table 1.

### Ethics

Ethics committee approval from both institutions was obtained prior to data collection.

### Endpoints

The primary endpoint was fracture localization and morphology in Vancouver B2 PFF, compared between short and standard stems. The secondary endpoints in the well-fixed nonfracture revision cohort were intraoperative femoral fracture during stem extraction, anatomic localization of extraction-related femoral damage, cerclage use, and the requirement for a modular or diaphyseal-engaging revision stem. Incision-to-suture time, estimated blood loss, transfusion, hemoglobin course, and in-hospital complications according to the Clavien–Dindo classification were reported as perioperative descriptive outcomes [18].

### Fracture pattern analysis

Fracture assessment was performed separately for the two clinical scenarios. In the Vancouver B2 cohorts, preoperative and perioperative radiographs were reviewed for the anatomic distribution of fracture involvement. In the well-fixed nonfracture cohorts, only fractures occurring during stem extraction were considered extraction-related events.

Fracture involvement was recorded for the greater trochanter, lesser trochanter, calcar region, and lateral cortex. Because a single fracture could involve more than 1 anatomic region, these categories were not mutually exclusive.

### Data analyses

Continuous variables are presented as means with standard deviations and categorical variables as counts and percentages. Statistical interpretation was organized by clinical scenario: short vs standard stems within the Vancouver B2 PFF cohort and short vs standard stems within the well-fixed nonfracture revision cohort.

Continuous variables were compared using independent-samples *t*-tests and categorical variables using chi-squared or Fisher’s exact tests, as appropriate. Comparisons between Vancouver B2 cases and well-fixed nonfracture revisions were considered descriptive and were not used to infer a stem-design effect because the indications and baseline clinical conditions differ fundamentally.

A *P* value of <0.05 was considered statistically significant (∗*P* < .05 and ∗∗*P* < .01).

All statistical analyses were run in GraphPad Prism 10.1.2 (GraphPad Software LLC, Boston, Massachusetts USA) and R Statistical Software (Version 2024.04.2 for macOS, R Core Team 2022, Vienna, Austria).

## Results

### Primary analysis: Vancouver B2 periprosthetic femoral fractures

The primary analysis included 56 Vancouver B2 PFF revisions: 44 standard stems and 12 short stems. Fractures around short stems showed more frequent involvement of the greater trochanter (7/12; 58.3%), lesser trochanter (4/12; 33.3%), calcar (5/12; 41.7%), and lateral cortex (7/12; 58.3%). In the standard-stem Vancouver B2 cohort, the corresponding frequencies were 18/44 (40.9%), 4/44 (9.1%), 6/44 (13.6%), and 14/44 (31.8%), respectively (Table 2, and Fig. 2a and b).

**Table 2:** Fracture localization, intraoperative fracture, modular diaphyseal reconstruction, and cerclage use across the 4 predefined cohorts.

Fracture localization	All	Standard-stem well-fixed nonfracture	Standard stem Vancouver B2 PFF	Short-stem well-fixed nonfracture	Short-stem Vancouver B2 PFF
	(n = 102)	(n = 27)	(n = 44)	(n = 19)	(n = 12)
Greater trochanter	34/102 (33.3%)	2/27 (7.4%)	18/44 (40.9%)	**7/19 (36.8%)**^a^	**7/12 (58.3%)**^b^
Minor trochanter	14/102 (13.7%)	0/27 (0%)	4/44 (9.1%)	**6/19 (31.6%)**^a^	**4/12 (33.3%)**^a^
Calcar	18/102 (17.6%)	2/27 (7.4%)	6/44 (13.6%)	**5/19 (26.3%)**^a^	**5/12 (41.7%)**^a^
Lateral cortex	27/102 (26.5%)	0/27 (0%)	14/44 (31.8%)	**6/19 (31.6%)**^a^	**7/12 (58.3%)**^a^
Preoperative	56/102 (54.9%)	0/27 (0%)	44/44 (100%)	0/19 (0%)	12/12 (100%)
Intraoperative	19/102 (18.6%)	4/27 (14.8%)	6/44 (13.6%)	**7/19 (36.8%)**^a^	2/12 (16.7%)
Postoperative	0/102 (0%)	0/27 (0%)	0/44 (0%)	0/19 (0%)	0/12 (0%)
Modular system usage	79/102 (77.5%)	13/27 (48.1%)	44/44 (100%)	10/19 (52.6%)	12/12 (100%)
Cerclage usage	78/102 (76.5%)	4/27 (14.8%)	43/44 (97.7%)	**10/19 (52.6%)**^a^	11/12 (91.6%)

Comparisons were made within the same clinical scenario (Vancouver B2 PFF or well-fixed nonfracture revision).

Bold represents statistical significance.

a Represents statistical significance, *P* < .05.

b Represents statistical significance, *P* < .01.

**Figure 2:**
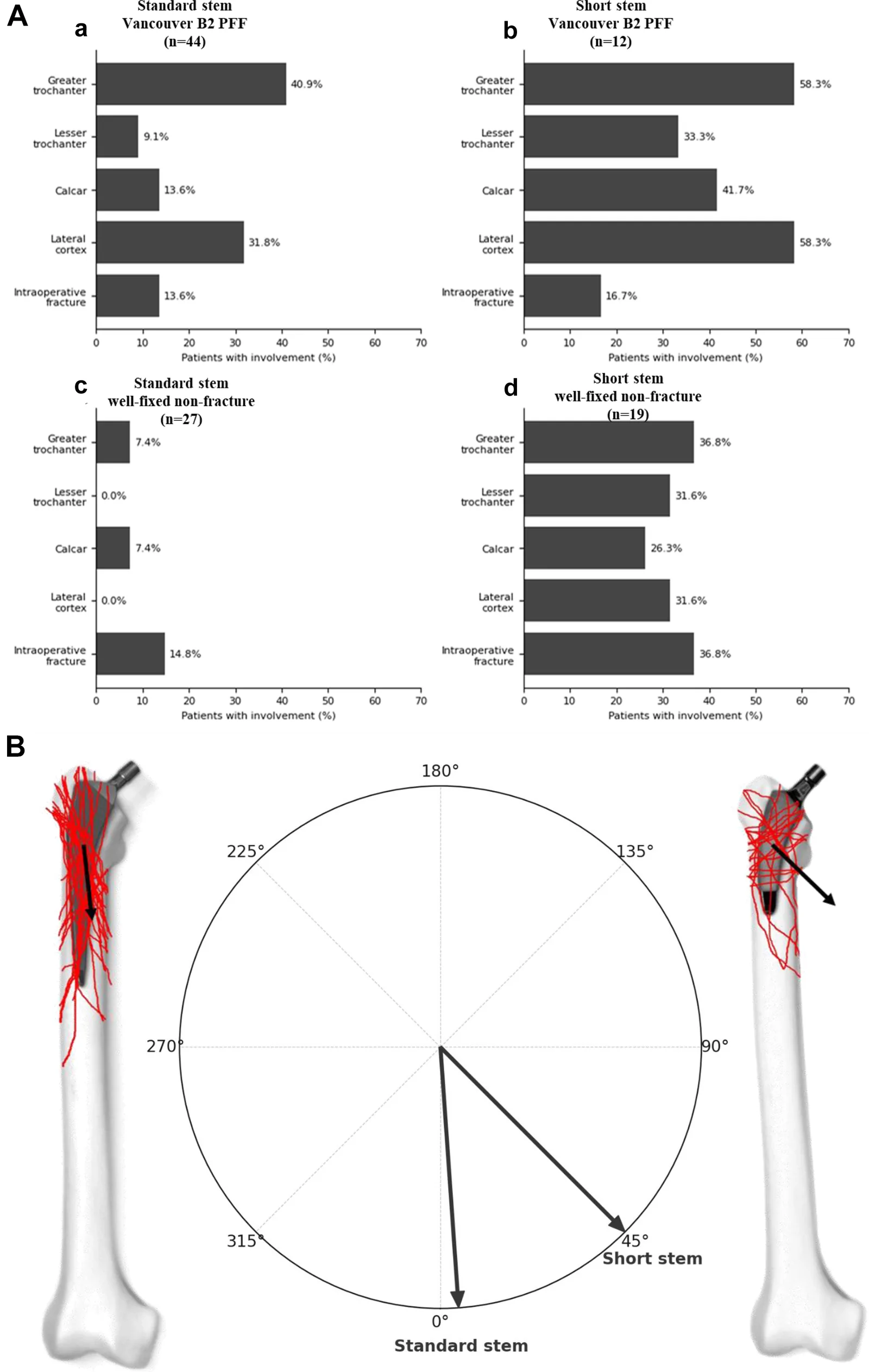
A) Cohort-specific distribution of fracture involvement. a) Standard-stem Vancouver B2 PFF (n = 44); b) short-stem Vancouver B2 PFF (n = 12); c) well-fixed standard-stem nonfracture revision (n = 27); d) well-fixed short-stem nonfracture revision (n = 19). Bars show the proportion of patients with involvement of the greater trochanter, lesser trochanter, calcar, lateral cortex. B) Fracture pattern of standard-stem and short-stem with red lines indicating fracture patterns and the main vector as a black arrow, further illustrated in a corona 360 circle.

The Vancouver B2 designation referred to the preoperative fracture indication and stem instability. During extraction, additional intraoperative fracture propagation was documented in 6/44 (13.6%) standard-stem and 2/12 (16.7%) short-stem Vancouver B2 cases. These extraction-related extensions were analyzed as additional events and were not pooled with the preoperative fracture localization when interpreting the primary fracture pattern.

All Vancouver B2 cases were reconstructed with modular revision stems providing diaphyseal anchorage. Cerclage fixation was used in 43/44 (97.7%) standard-stem and 11/12 (91.6%) short-stem Vancouver B2 revisions (Table 2, Fig. 3b-d).

**Figure 3:**
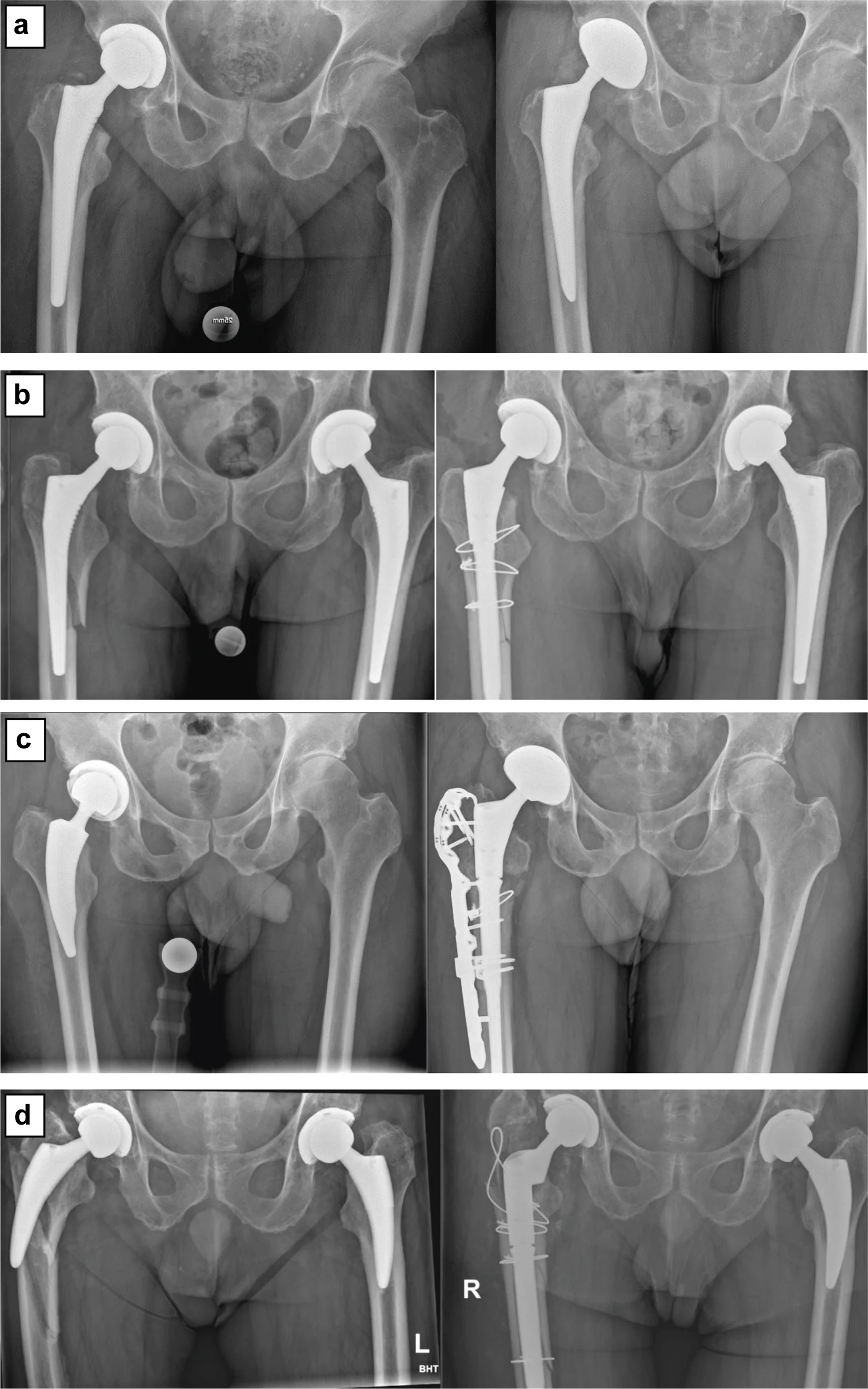
Representative anteroposterior radiographs from each of the 4 cohorts. a) Well-fixed standard-stem nonfracture revision. b) standard-stem Vancouver B2 PFF revised to a modular diaphyseal stem with cerclages. c) well-fixed short-stem nonfracture revision with extraction-related femoral damage and cerclage fixation. d) short-stem Vancouver B2 PFF revised to a modular diaphyseal stem with cerclage fixation.

### Secondary analysis: well-fixed nonfracture stem revisions

The secondary analysis included 46 well-fixed, osseointegrated stems revised for nonfracture indications: 27 standard stems and 19 short stems. Intraoperative femoral fracture during extraction occurred in 4/27 (14.8%) standard-stem revisions and 7/19 (36.8%) short-stem revisions. In the short-stem group, extraction-related damage involved the greater trochanter in 7/19 (36.8%), lesser trochanter in 6/19 (31.6%), calcar in 5/19 (26.3%), and lateral cortex in 6/19 (31.6%); the corresponding frequencies for standard stems were 2/27 (7.4%), 0/27 (0%), 2/27 (7.4%), and 0/27 (0%) (Table 2, and Fig. 2a and b).

Cerclage fixation was required in 10/19 (52.6%) well-fixed short-stem revisions compared with 4/27 (14.8%) well-fixed standard-stem revisions. Modular diaphyseal reconstruction was used in 9/19 (47.4%) short-stem and 8/27 (29.6%) standard-stem revisions (Fig. 3). Difficult short-stem extraction was particularly observed when a curved stem showed distal osseointegration or valgus positioning in a Dorr type B femur (Fig. 4).

**Figure 4:**
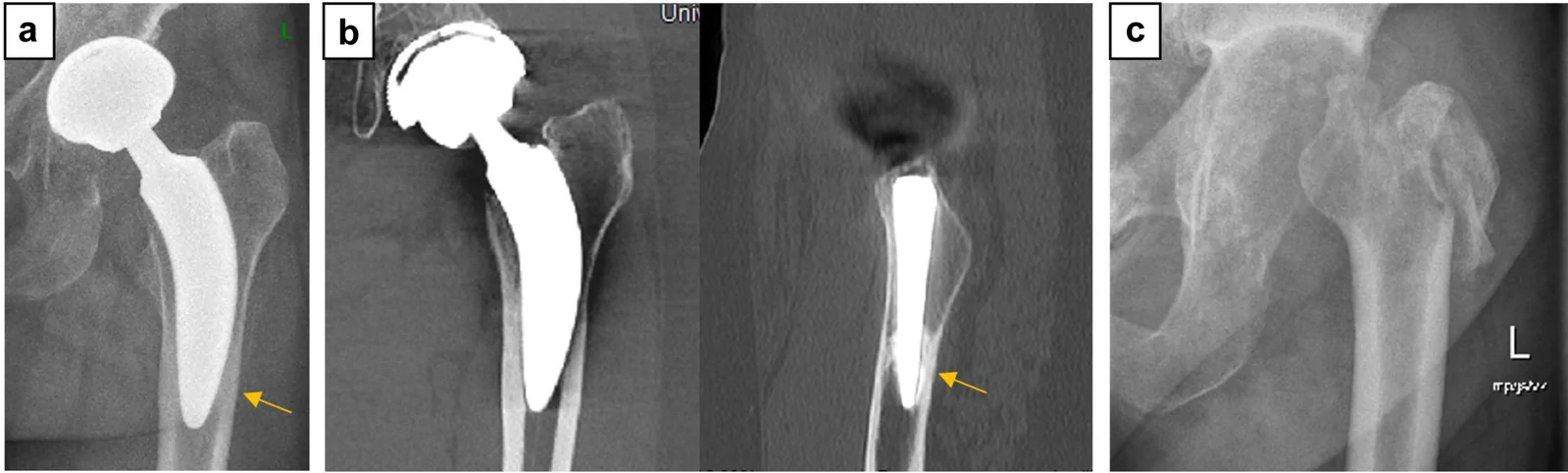
(a-c): Anteroposterior radiograph and coronal/sagittal computed tomography images of a well-fixed short stem with valgus position and distal osseointegration (arrow), followed by the postoperative radiograph after difficult extraction with trochanteric fracture. The case illustrates how stem geometry, position, and the location of osseointegration may increase extraction complexity.

### Descriptive perioperative outcomes across the full cohort

Across all 102 revisions, cerclage fixation was required in 78 cases (76.5%) and a modular revision stem with diaphyseal anchorage in 73 cases (71.6%). These pooled values describe the overall surgical burden but were not used to compare the two clinical indications.

Perioperative complications occurred in 76/102 (74.5%) patients; 51/76 (67.1%) were classified as nonsurgical and 25/76 (32.9%) as surgical complications.

Mean incision-to-suture time was 160.5 ± 46.4 min. Operative time was longer in the Vancouver B2 cohorts than in the well-fixed nonfracture cohorts, consistent with the different clinical scenarios; these between-scenario differences were interpreted descriptively only.

Mean estimated blood loss was 1059 ± 570 ml. Estimated blood loss was 821 ± 454 ml in well-fixed short-stem revisions and 1100 ± 334 ml in short-stem Vancouver B2 revisions. Because estimated blood loss is influenced by fracture severity, surgical exposure, and surgeon-specific estimation, it was considered a supportive rather than definitive measure of procedural complexity.

Overall, 54/102 (52.9%) patients received a blood transfusion. Transfusion was required in 10/27 (37.0%) well-fixed standard-stem revisions, 8/19 (42.1%) well-fixed short-stem revisions, 26/44 (59.1%) standard-stem Vancouver B2 revisions, and 10/12 (83.3%) short-stem Vancouver B2 revisions (Table 3).

**Table 3:** Descriptive perioperative outcomes across the 4 cohorts.

	All	Standard-stem well-fixed nonfracture	Standard-stem Vancouver B2 PFF	Short-stem well-fixed nonfracture	Short-stem Vancouver B2 PFF
	(n = 102)	(n = 27)	(n = 44)	(n = 19)	(n = 12)
Incision-suture time (min)	160.5 ± 46.4	130.5 ± 24.4	179.2 ± 45.8	143.0 ± 49.2	176.2 ± 43.6
Estimated blood loss (ml)	1059 ± 570	1119 ± 676	1134 ± 583	821 ± 454	1100 ± 334
Transfusion	54/102 (52.9%)	10/27 (37%)	26/44 (59.1%)	8/19 (42.1%)	10/12 (83.3%)
Clavien–Dindo Classification					
0	26	10	7	7	2
1	9	3	5	1	0
2	40	9	19	5	7
3a	2	0	2	0	0
3b	13	3	6	3	1
4a	6	1	2	2	1
4b	3	0	2	0	1
5	3	1	1	1	0

ml, milliliter; min, minutes.

The complete in-hospital complication profile according to the Clavien–Dindo classification is shown in Table 3.

## Discussion

First, among Vancouver B2 PFFs, short stems showed a more proximally concentrated pattern of femoral involvement, particularly around the trochanters, calcar, and lateral cortex. Second, among well-fixed stems revised for nonfracture indications, removal of short stems was frequently accompanied by extraction-related proximal femoral damage and a greater need for cerclage fixation. These findings should be interpreted within each clinical scenario rather than by comparing traumatic PFF directly with elective/nonfracture revision.

The primary Vancouver B2 analysis supports the concept that implants geometry and fixation pattern may influence the “personality” of a periprosthetic fracture. Short stems depend predominantly on metaphyseal fixation and proximal load transfer, whereas standard stems generally provide additional metaphyseal-diaphyseal engagement. Differences in local stress transfer may therefore contribute to the distinct anatomic distribution of fracture involvement observed in the present cohort.

Importantly, the fracture-pattern findings in Vancouver B2 cases are distinct from the secondary observation of iatrogenic fracture during extraction. A B2 fracture is present before revision and is associated with stem instability, whereas an extraction-related fracture develops during removal of a previously well-fixed stem.

In the well-fixed nonfracture revision cohort, intraoperative fracture occurred in 36.8% of short-stem removals compared with 14.8% of standard-stem removals. Short-stem extraction also required cerclage fixation in 52.6% of cases. These findings suggest that a short implant is not necessarily synonymous with an easy revision once substantial osseointegration has developed.

The growing popularity of short-stem THA is largely based on theoretical and demonstrated advantages during primary implantation, including bone preservation, proximal load transfer, and reduced stress shielding [[19], [20], [21]–22]. These benefits at the index operation, however, should be distinguished from the technical demands of later removal.

Previous reports of relatively straightforward short-stem revision have often included stems revised for loosening, malposition, or other situations in which fixation may already be compromised [16,23]. In contrast, the present secondary cohort specifically focused on stems that were mechanically well fixed before extraction. In this setting, the location and extent of osseointegration may be more relevant to extraction difficulty than stem length alone.

The need for modular diaphyseal reconstruction in 47.4% of well-fixed short-stem revisions indicates that proximal bone preservation at primary implantation does not guarantee preservation of the same bone stock at revision. Nevertheless, this finding should not be interpreted as proof that short stems are inferior to standard stems; the groups were small, retrospective, and differed in patient characteristics and revision indication.

The case illustrated in Figure 4 provides a mechanistic example: distal osseointegration of a curved short stem in valgus position made the fixation zone difficult to access and was associated with trochanteric fracture during extraction. Such implant- and anatomy-specific factors may be particularly important when planning removal of metaphyseal short stems.

Biomechanical and clinical literature supports the broader principle that stem geometry affects proximal femoral loading and fracture behavior [9,10,21,24]. The present study extends this concept to revision surgery by showing that the relevant failure pattern depends not only on stem design but also on whether the patient presents with an established Vancouver B2 fracture or with a well-fixed stem that must be removed for another indication.

The high rates of cerclage use and modular diaphyseal reconstruction in the Vancouver B2 groups primarily reflect the fracture indication itself and should not be interpreted as evidence that 1 stem type is more difficult to revise in this setting.

Conversely, the well-fixed nonfracture comparison provides the more appropriate setting in which to evaluate extraction-related burden. Here, the numerical differences in intraoperative fracture and cerclage use favor caution when assuming that metaphyseal short stems will always be easier to remove than standard cementless stems.

Perioperative morbidity remained substantial across the overall cohort. However, the complication burden is strongly influenced by the underlying revision indication, particularly the acute trauma and physiologic stress associated with Vancouver B2 PFF. Therefore, overall complication and transfusion rates are presented descriptively and are not used as direct evidence of a stem-design effect.

For clinical practice, the findings emphasize preoperative identification of the fixation zone, stem geometry, proximal femoral morphology, and potential need for cerclage or diaphyseal reconstruction. In well-fixed short stems, surgeons should anticipate that proximal osseointegration can require a more extensive extraction strategy than the implant length alone might suggest.

A prospective series of 45 failed short-stem revisions reported that no trochanteric osteotomies or femoral windows were required and that conversion to standard stems was frequently possible [23]. This apparent difference is not necessarily contradictory because the indication and fixation status at revision are critical: a loose or incompletely integrated short stem is fundamentally different from a well-osseointegrated stem requiring deliberate disruption of the bone–implant interface.

Accordingly, future studies should stratify revision cohorts by both stem design and fixation status rather than by stem length alone. This would allow a clearer distinction between implant failure patterns, fracture morphology, and extraction-related complications.

The same principle applies to periprosthetic fracture research. Future computed tomography-based studies should assess three-dimensional fracture morphology, bone stock, stem position, and the spatial relationship between the fracture line and the fixation zone. Such analyses may help determine whether the proximal fracture pattern observed around short stems is reproducible across implant designs.

The present data do not support the conclusion that standard stems are inherently easier to revise. Rather, standard and short stems appear to create different mechanical environments. Additional diaphyseal engagement in standard stems may shift fracture involvement distally, whereas strong metaphyseal fixation in short stems may concentrate stress in the proximal femur.

This distinction is particularly relevant as short-stem THA is increasingly used in younger patients who may face revision during their lifetime. Implant selection for primary THA should remain based on established indications and outcomes, while revision planning should account for the fixation characteristics of the specific stem.

To our knowledge, few clinical studies have directly described both the anatomic fracture distribution around contemporary short stems and the extraction-related consequences of removing a well-fixed short stem. The present two-center cohort therefore provides hypothesis-generating data for two related but analytically separate aspects of revision THA.

This study has several limitations. Its retrospective design and relatively small subgroup sizes limit statistical power and causal inference. Surgical approach and extraction strategy were not standardized across the 6 surgeons, reflecting real-world practice but introducing procedural heterogeneity. Not all patients underwent computed tomography imaging. Therefore, fracture assessment was based primarily on two-dimensional radiographs. The mechanism of the index traumatic fracture was not consistently available. In addition, the study contains 4 clinically distinct subgroups and should be considered exploratory. Larger multicenter cohorts with standardized three-dimensional imaging and subgroup-specific analysis are required to validate the observed patterns.

## Conclusions

This study demonstrates that short and standard cementless stems are associated with different patterns of femoral involvement in Vancouver B2 PFF. In a separate analysis of well-fixed nonfracture revisions, short-stem extraction was frequently associated with proximal femoral damage and additional cerclage fixation. The results emphasize that periprosthetic fracture morphology and extraction-related complications represent different clinical phenomena and should be analyzed separately when evaluating revision characteristics of short and standard stems.

## Data availability

The data that support the findings of this study are available from the corresponding authors upon reasonable request.

## Ethics approval

This study was performed in line with the principles of the Declaration of Helsinki. The approval of the ethics committee was obtained prior to the experiment (Ethikkommission der Medizinischen Universität Graz, reference number EK-Nummer: 34-400 ex 21/22, Ethikkommission der Medizinischen Fakultät der Ludwig-Maximilians-Universität, reference number: 25-0037).

## Funding

No financing was received for this study.

## Conflicts of interest

The authors declare no potential conflicts of interest.

For full disclosure statements refer to https://doi.org/10.1016/j.artd.2026.102150.

## CRediT authorship contribution statement

**Dominic Simon:** Writing – review & editing, Writing – original draft, Visualization, Methodology, Formal analysis, Conceptualization. **Gautier Beckers:** Writing – review & editing, Writing – original draft, Methodology, Conceptualization. **Florian Pachmann:** Writing – review & editing. **Amir Koutp:** Writing – review & editing. **Salome Hagelstein:** Writing – review & editing. **Eduardo Suero:** Writing – review & editing. **Boris M. Holzapfel:** Writing – review & editing, Supervision. **Lukas Leitner:** Writing – review & editing, Writing – original draft, Supervision, Project administration, Formal analysis, Conceptualization.

## Appendix A Supplementary data

Conflict of Interest Statement for Holzapfel

Conflict of Interest Statement for Koutp

Conflict of Interest Statement for Leitner

Conflict of Interest Statement for Hagelstein

Conflict of Interest Statement for Simon

Conflict of Interest Statement for Suero

Conflict of Interest Statement for Suero

Conflict of Interest Statement for Beckers
